# Ethical Practices in Dentistry: Call for Bioethics Education and Collective Action

**DOI:** 10.12669/pjms.41.3.11907

**Published:** 2025-03

**Authors:** Afifa Ehsan, Ali Raza

**Affiliations:** 1 Prof. Dr. Afifa Ehsan BDS, MPhil, CMT, CME, CMJE, DHEP, MHPE, PhD Scholar Professor, Department of Oral Biology, Faryal Dental College, Lahore, Pakistan; 2 Prof. Dr. Ali Raza BDS, M.Phil, PGD (Bioethics). University Of Health Sciences, Lahore, Pakistan

The word “Ethics” is derived from the Greek word “*ethos*” which means “character or conduct”. The American College of Dentists (ACD) has defined ethics as “moral principles or virtues that govern the character and conduct of an individual or a group”. 1-2 The origin of the field of ethics is attributed to the subjects of philosophy and theology. The roots of medical ethics can be traced back to Hippocrates (400-300 BC). However, appalling medical events after the second world war gave rise to contemporary medical ethics and bioethics.^3^ There is a general accord that bioethics emerged in the United States and the Western world, and their cultural influences can be seen in the current ethical discourses.^4^

Medical Ethics have become a pivotal constituent of medical education in the developed world. The World Health Organisation (WHO) guidelines for teaching medical ethics (1994) have remarkably drawn an analogy of medical ethics to the human body. It has portrayed learning the science of medical education as being ‘head’, gaining psychomotor skills representing ‘hand’, and knowledge of professionalism and medical ethics as ‘heart’.^5^ There are several ethical issues that healthcare professionals encounter in their routine practices. Some of the common ethical issues include confidentiality of the patients’ information, informed consent before offering possible treatment options, making evidence-based decisions, and sanctity of the doctor-patient relationship.^3^ The International Dental Federation (FDI) has published “International Principles of Ethics for the Dental Profession” (2007). According to these guidelines, “the professional dentist will practice according to the art and science of dentistry and the principles of humanity, and will safeguard the oral health of patients irrespective of their status”.^6^

Although the Pakistan Medical and Dental Council incorporated the Code of Ethics in medical/dental curricula in 2004 and urged medical/dental institutions to implement it, yet, it mostly remains an unfulfilled goal.^7^ Aga Khan University Medical College, Karachi incorporated biomedical ethics in their undergraduate curriculum in 1984. The first research ethics committee came into being with the foundation of the Pakistan Journal of Medical Ethics in 1997, followed by the establishment of the National Bioethics Committee (NBC) in 2004.^7^-^9^ Institutional Review Boards (IRBs) were appointed later to enhance ethical aspects of medical/dental research including obtaining written informed consents from participants, ensure the confidentiality of personal information collected and subsequent utilization of the information gathered.^7^,^9^ In its stipulations, PM&DC (2001) emphasized the inclusion of biomedical ethics in the medical curriculum.^9^

A dynamic step was taken in 2004 by founding the Centre of Bioethics and Culture (CBEC) at the Sindh Institute of Urology and Transplantation (SIUT), Karachi.^9^ It launched a Postgraduate Diploma Program in Biomedical Ethics (2006) followed by a Master of Bioethics Program in 2009.^10^ It also introduced certificate programs in research and clinical ethics in 2014. Furthermore, Shalamar Medical & Dental College has recently developed a Department of Bioethics offering diploma programs.

Currently, both individual as well as institutional efforts are being made to sensitize healthcare professionals about ethical practices.^8^ CBEC alumni; the torchbearers of bioethics, have commenced a chain of activities related to ethical issues nationwide.^11^ Ethical grand rounds, lecture series, bioethics groups, institutional review boards, ethical review committees, research ethics training programs, and bioethics forums are some examples of these efforts.^12^ Alas, bioethics education is not yet a regular component of the medical/dental curriculum in Pakistan and is taught haphazardly except in a few institutions compared to the developed countries where bioethics is mandatory in medical and dental schools.^3^

Like other healthcare professionals, the dental fraternity also comes across many ethical challenges during their clinical practices and other professional activities.^13^ A framework is needed to guide these professionals in their daily dental practices as per the local socio-cultural norms. The Codes of Ethical conduct have been developed by dental organizations worldwide; the American Dental Association developed its Code of Ethics in 1866.^1^ However, the journey of ethical guidelines in dentistry has yet to gain momentum in Pakistan. The dental curricula lack bioethics as a regular component. Dental students are future dental clinicians, academicians, and researchers. In either role, they are exposed to a series of ethical issues. To cope with these ethical challenges, they must be provided with a strong basis for making ethical decisions.^14^

As one reflects on the condition of medical ethics in Pakistan, it becomes apparent that the time for concerted action is ripe. The dental fraternity, like its medical counterpart, stands at crossroads, struggling with ethical dilemmas that mandate meticulous contemplation and well-versed decision-making.^15^ Devoid of a strong background knowledge of ethical practices, dental professionals are left susceptible to hesitation and moral indistinctness. In the search for ethical excellence, there is a joint accountability to encourage the inclusion of bioethics education in medical and dental curricula and enable future generations of healthcare professionals with the understanding and skill needed to steer multifaceted ethical landscape.^15^ It is only through the combined efforts, grounded in a pledge to ethical integrity and patient-centered care, we can chart a course where ethics serve as the beacon of healthcare practice in Pakistan and beyond.

Focusing on the ethical challenges faced by medical and dental professionals in Pakistan needs a comprehensive methodology targeted at coaching, making guidelines, and bringing about a social transformation. A significant resolution is the required amalgamation of bioethics education into medical and dental curricula, making sure that forthcoming specialists are prepared with the knowledge and skills required to pilot multifaceted ethical dilemmas. This teaching must be inclusive, merging knowledge with case studies, promoting critical thinking, and aiding moral reasoning. The formation of regulatory bodies with the authority to implement ethical guidelines and deliver continuous professional development is also crucial. Nevertheless, the accomplishment of these resolutions faces barriers, including the struggle to bring about curriculum transformations, the deficiency of qualified bioethics professionals, and the deep-rooted cultural traditions that may contradict ethical guidelines. Furthermore, deficiency of resources and institutional provision causes delays. Conquering these trials needs a combined struggle by educational institutions, professional bodies, and government to generate an atmosphere where ethics is highlighted and strengthened at all levels of healthcare practice.

## Authors’ Contribution:

**AE** wrote the first draft of the article which was subsequently revised by **AR**.

Both authors approved the final submission.
